# Implementation of the ABCDE bundle: results from a real-world, pragmatic study design

**DOI:** 10.1186/1748-5908-10-S1-A3

**Published:** 2015-08-14

**Authors:** Andrew Masica, Ashley Collinsworth, Maria Kouznetsova, Candice Berryman, Sophie Lopes, Susan Smith

**Affiliations:** Center for Clinical Effectiveness, Baylor Scott & White Health, Dallas, TX 75206 USA; Baylor University Medical Center, Baylor Scott & White Health, Dallas, TX 75246 USA

## Introduction

Care processes in the ABCDE bundle (daily awakening, breathing trials, delirium management, early mobility) have been shown to improve a range of clinical outcomes in intensive care unit (ICU) patients. However, uptake of the ABCDE bundle has been inconsistent to date. We examined the effectiveness of a structured implementation program on ABCDE bundle adoption across ICUs in 6 different Baylor Scott & White Hospitals.

## Methods

We conducted the study under a quasi-experimental design in 2 different intervention groups ("basic" and "enhanced", 3 hospitals per group) with time series analyses. All hospitals had access to modified EHR documentation to facilitate ABCDE process capture. Additional tactics at enhanced intervention hospitals involved site champion engagement, supplemental bundle training, and participation in development of ABCDE performance reports. We assessed multiple time points to evaluate overall effectiveness of control versus intervention practice adoption approaches as well as impacts of individual components in the implementation program.

## Results

Analysis cohort included 979 patients (330 in Year 1, 649 in Year 2). Hospitals in both intervention groups showed statistically significant improvements in adherence to individual components (15%-49% absolute increase, P < 0.05) and the composite bundle (15%-22% absolute increase, P < 0.001) between years. Enhanced intervention hospitals demonstrated practice uptake earlier in Year 1, but the quarterly rate of adoption was 2-fold higher in basic intervention hospitals (m = 0.06 vs 0.03), which had a drastic increase in bundle adherence (16% vs. 2%) in the 3 months following EHR modification. Composite bundle performance between groups converged by Year 2 end.

## Conclusions/Advancement to dissemination and implementation science

ABCDE bundle performance improved with use of structured implementation programs. Modification of

EHR workflow and documentation accelerated ABCDE practice adoption at a faster rate relative to traditional implementation strategies. Focusing resources on EHR modification (and placing this phase as early as possible in the implementation program sequence) appears to be a high-yield practice uptake approach.

This project was supported by a grant (R18HS021459) from the Agency for Healthcare Research and Quality (AHRQ). The findings and conclusions in this document are those of the authors, who are responsible for its content, and do not necessarily represent the views of AHRQ.

